# Paralysis and Convulsions as Effects of Diseases of the Base of the Brain

**Published:** 1878-04

**Authors:** 


### PARALYSIS AND CONVULSIONS AS EFFECTS OF DISEASES OF THE BASE OF THE BRAIN.

Delivered in Chicago February 21st, 22d and 23d, 1878.

By Dr. Brown-Séquard.

*(Reported for the Journal and Examiner.)*

SECOND LECTURE.

Gentlemen: In the last lecture (the first of this course), I have tried to show that a decussation of the nerve fibers in the base of the brain, although we know it to exist anatomically, is not the least essential for the communication between the brain and the muscles of the body. For the present, continuing the demonstration which I began in the first lecture, I have to speak of the crura cerebri. You remember I told you that a lesion in one-half of the angle in the crura cerebri and medulla oblongata, may take on various symptoms as regards paralysis, or no paralysis at all. I also said that a lesion occupying half the pons varolii either produces no paralysis or produces a great variety of paralysis as regards its extent and seat. The same thing can be said as regards the crura cerebri. A lesion destroying the greater part of the crura cerebri, can produce the greatest variety of symptoms as regards paralysis and convulsions. And, indeed, it is a remarkable fact that any such mass of fibres as those constituting the crura cerebri, forming the only communication between the cerebral lobes, the spinal cord, the pons varolii and the medulla oblongata—that such a mass of fibers, establishing, as I have said, the only communication between the center of will-power and the muscles, can be destroyed almost entirely without

23

any marked paralysis. I must say that when I first learned this fact I was exceedingly surprised. But since then I have gathered from journals, from books and from my own experience, cases until now I have more than 27 of more or less complete destruction of one of the crura cerebri, without any marked paralysis in the limbs. In all these cases there were symptoms which pretty clearly showed the place of lesion. But as regards paralysis there were none—at least, none were known by the medical men who observed the patients, and the main fact remains that there was nothing of that gross paralysis which we observe frequently arising from some slight lesion in some part of the surface of the brain, the medulla or posterior lobe of the brain. So that we can say that, with the destruction of tissue, which is considerable in that part which is the only channel of communication between the brain and the muscles, there has been very little paralysis, if any ; and, indeed, none was noticed in most cases. On the contrary, paralysis has appeared on the side corresponding with that of the lesion. This fact is in opposition with what is admitted. In other cases, paralysis has appeared where it ought to come in all cases, admitting theories we have received to be true.

But in these cases, if you study the facts with care, you find the greatest discrepancies with the same lesion. The paralysis of the two limbs on the opposite side, may be caused by disease of the crura cerebri. Such paralysis may come from only lesion of a very small part of the crura cerebri, leaving most of it perfectly normal. And there you have really too much effect to support the theories which are generally admitted. So that in any way you turn the facts, in almost all cases, you find some figures in opposition to the views admitted. Either there is too much paralysis, or no marked paralysis, or paralysis exists where it should not, in the arm or leg or indefinitely, although the lesion is in the same part in the various cases. Such variety of effect certainly could not exist if the views admitted were true.

I pass now to another argument. One of the most interesting of facts relating to paralysis in brain disease, consists in its coming by attacks, just as convulsions come. There are a number of cases in which paralysis has come and gone — come for an hour, for a day, for two days, and then disappeared altogether, and then has returned for a certain length of time and disappeared again. In a large number of these cases, I have concluded that the disease was in the crura cerebri, the pons varolii or the medulla oblongata. Now, if you admit that these are the only channels by which the will power can act upon the muscles upon one side of the body, you are in the presence of facts in opposition to your views. How can it be, if these fibers are destroyed, that the paralysis exists only for a few hours, and then disappears? Certainly we cannot admit that paralysis, in these cases, depends upon the destruction of the tissue establishing communication between the brain and the muscles. We must look for another reason in these cases, as well as in many others.

Another argument, of more importance, on which I should like to dwell at length, consists in the fact that in cases of disease occupying the central part of the pons varolii, and destroying the nerve fibers, not only of the center, but of the front part of the two sides of the middle line, it is not rare that instead of paralysis on the two sides of the body, as should exist if the views admitted by almost everybody are true, there is nothing but hemiplegia, that is, paralysis on one side of the body. This argument is certainly most weighty: and, indeed, the cases are not to be doubted as regards the accuracy of the observer and the details, for they come from men in whom we have confidence, and among the writers there are two, certainly, so well known that there cannot be the least doubt as regards their accuracy or their facts — Frederick Charcot and Professor Eugène, of the French Medical School. So that we have facts which show clearly that with a similar lesion on the two sides of an organ like the pons varoli, which is the only way through which fibers coming from the brain go to the muscles, instead of producing paralysis on both sides, produces only paralysis on one side, which is hemiplegia.

After having mentioned these facts, it is quite essential that I should say something which perhaps would have been well placed in the beginning of the last lecture, which is, that the most considerable variety of effects can be observed from similar lesions in the brain of man. It is in that variety of effects that we find the reason explaining why we see in one individual a paralysis on the same side from a certain lesion, and in another individual paralysis on the opposite side ; in a third, paralysis on the two sides, and in a fourth no paralysis at all ; and so on, giving the greatest variety not only as regards paralysis, but as regards convulsions. This variety, in effect, indicates, as I conceive, that the cause of that variety depends on the various excitability of different parts of the nervous system. As you well know, the nervous system is a unity in such a way that no action can take place in one part without the whole mass being acted upon in some measure ; in the same manner that, while I am only an old man having very little muscular strength, I can shake the whole universe by the movement of my foot. If such a shock, slight as it is, can produce (as no one who knows about the transmission of motion can deny) a shock through the whole universe, can disturb everything in the universe, in a very slight way it may be, in the same way any action in any part of the nervous system produces some effect in every other part, however distant it may be. If so (and I wish I could insist upon the demonstration that it must be so, but I have no time, unfortunately) we must admit that in this case it might be so. In case the excitability of a certain part is greater than that of another, the excitation will find that particular part excitably disturbed far more than other parts, and morbid phenomena will occur in that part. If you observe what takes place when a large mass of people come from a theater or any other large room where the temperature is high, you will find that perhaps 150 will escape without meeting any bad effects from passing from the warm room into the damp, cold air, while perhaps 50 will be affected. Out of the 50 there will hardly be more than a few who will experience exactly the same effect. In all these individuals so affected, however, the same cause will have operated. In some cases cold air on the neck will make itself felt. In others it will exert its effects on the trachea, the bronchial tubes, or the skin. From pretty much the same cause, therefore, there will be a number of individuals who will have trouble with the secretions, or perhaps with the mucous membrane of the throat, or the larynx, the trachea, the bronchial tubes, the lungs, the pleura, the heart, or the pericardium. Others, on the contrary, will experience the effects of the cold air in the bladder, the kidneys, the stomach, the bowels or liver. Any organ, in fact, in the system, may be acted upon through the same cause in most of these individuals. Even the ear, the brain, or any part of the body liable to be inflamed, may be acted upon by inflammation from such a cause. Well, if we see such variety from causes pretty much the same, we can easily understand that when hemorrhage takes place in the brain, all the various symptoms of paralysis may arise. On this point there are a great many interesting cases that I might bring forward; but there is one published by Dr. Campbell which is most interesting. A boy ten years old was seized with vomiting. Immediately after vomiting he was seized with general convulsions of the muscles of the face, neck and eyes. In a very short time a contraction took place in the blood vessels of the trunk and lower limbs, so that intense cold appeared in these parts. The pupils then contracted, and a very short time afterwards considerably dilated. The pulse became weak and small, and in twelve hours the patient died. As is usual in these cases, there were difficulties of breathing and circulation. What had caused all that? A small hemorrhage—a clot being found no larger than half a walnut, and located in one of the convolutions of the middle lobe on one side. If we were to look upon its action as having taken place according to the theory now admitted, it would have acted upon a series of centers, a center of dilatation and a center for contraction ; it would have acted upon a center in communication with the muscles of the body. It would have acted also upon the stomach and other muscles of the abdomen. It would have acted in fact upon any part of the pretended centers ; upon the center to arrest the heart's action, or to diminish it. We certainly cannot admit that such a series of centers can exist in a small part of the brain, but must admit the great variety in the excitability of different parts. Irritation starting from a small part of the brain, and finding some parts more excitable than others, will produce diverse effects. In that way we have a key to all these phenomena quite easy to understand.

Then, as you well know, the irritation of taenia may produce convulsions, paralysis, catalepsy, chorea ; indeed every symptom that diseases can produce, when they exist in the brain, may appear through such peripheric irritation; in the same way (as I have tried to show in an article on surgery), a nerve, by traumatic action, can produce the greatest variety of effects—paralysis, convulsions, and so on. So that, as you see, great differences of excitability in certain parts of the nervous system in different individuals, will create a great variety of effects. It is in this way that we can explain the excessive variety that diseases of the brain can produce, although existing in the same part of the brain in the various cases. Experiments consisting in burning with a white-hot iron the surface of the brains of animals, show very clearly how various are the effects of the same cause. In a great many of these animals rendered anæsthetic for a time by the injection of chloral, so as to avoid suffering, cauterization was made without the least pain. In these cases the variety of effects was impossible. In some cases there are phenomena resembling exactly those which we find in man and animals, when there is meningitis of the spine as well as of the spinal cord itself. Thus, in meningo-myelitis these phenomena appear, and appear so as to come on the two sides, while the lesion is only on one side. In one case the animal was shown to my colleague in Paris, who exclaimed, "Why, in the world, do you show us this dog; it is a dog with meningo-myelitis; what is your object?" I said, my object is to show you that there is no such thing whatever. There had been no injury to the spinal cord, and there was only a slight degree of congestion in parts of the cord, and no inflammation whatever; but still all the phenomena of meningo-myelitis existed which had been noticed by inflammation starting from the surface of the brain. In most cases of paralysis the sympathetic nerve on the same side is affected, so that the eye, the face and the ear, exhibit all the phenomena that will appear after the division of a nerve. So you see, we can find by experiment a clear demonstration that the same lesion, such as we can make in animals, will produce the most various effects.

This leads me to return now to the various points which I said I intended to establish in this course of lectures. At first I will speak of the influence that one-half the brain can exert on the body. As you well know, the theories which all have admitted, and most men still admit, imply that the right half of the brain is the organ that moves the left side of the body. I have tried to show, up to this time, and will attempt the complete demonstration in this lecture and the next, that on the contrary one-half the brain may be perfectly sufficient for the functions that belong to the two sides as regards the moving of the muscles of the body. The first argument on which I will again insist as most decisive of all to establish the fact that one-half the brain is sufficient, is that a lesion existing in any part of half the brain may produce no paralysis whatever, or at any rate no marked paralysis. If you take any of the various parts of the brain, the anterior, middle, or posterior lobe, and divide one hemisphere, say at the temporal or occipital lobes, you will produce a lesion without any marked paralysis. Still more, there are cases in which several of these lobes have been destroyed without marked paralysis. And there are a number of cases (not more than four or five however), in which almost the whole extent of one hemisphere has been destroyed without any marked paralysis. One case of that kind was reported by Dr. Abercrombie, and one was published in one of the numbers of last year of my own physiological journal, and one in the Archives of Physiology, published in Paris, the fact having been recorded by a physician of Italy. But these cases are all of less significance than cases in which the disease exists in one-half of the base of the brain without any marked paralysis, for we may say if half the upper part of the brain, or if the lobes that form one hemisphere are destroyed, that the base is able to perform its functions; but when the base is destroyed in one of its parts, so that no communication can exist between one-half of the lobes and the opposite half of the brain, it is certain that such a case is more conclusive than any where the whole hemisphere, excepting the base, has been completely destroyed. If you have taken for granted what I said in the lecture yesterday, you will see that there are a number of cases in which disease may exist in one-half the pons varolii, the medulla oblongata, and the crura cerebri, where there may be destruction of the parts on one side without any marked paralysis. Therefore it is quite evident, if these cases exist (and if I had time I could mention them in detail, but they are soon to be published, when you can look at their details), nothing remains but the conclusion that one-half of the brain acts upon either side of the body. It is clear, therefore, that the commonly adopted view is wrong, and that we must admit that one-half the brain is sufficient for the movements of the two sides of the body. It remains, however, to examine why paralysis appears in cases in which there is a lesion, and this point I will try to illustrate in my next lecture.

There are other arguments that establish that half the brain is sufficient for the movements of the two sides of the body. If we find, for instance, that disease in one-half the base of the brain produces paralysis on the corresponding side, unless we admit that some movements are constituted quite distinct from others as regards the anatomical disposition of the tissues, we must admit that this paralysis is due to the ordinary causes of paralysis, according to admitted notions. It is quite certain that what is admitted is, that one side governs the muscles of the opposite side. If so, the fact that disease on one side of the brain may produce paralysis on the corresponding side of the body is clearly in opposition to the admitted theory.

I will pass—as I am compelled to do so for lack of time—from a good many arguments bearing on this point, and come to another point of greater importance, which is that a few fibers may be sufficient to establish a communication between the brain and the muscles in the various parts of the body. When I say a few fibers, I mean relatively a few; I will not say how many, but certainly a very limited number of fibers is sufficient for the transmission of the orders of the will to the muscles. Cases are numerous, in which the base of the brain, particularly the medulla oblongata, has been so pressed upon that a very small number of fibers has remained in these parts able to transmit any order of the will from the brain to the muscles. Take the case like that of the sailor who had been continuing the duties which such men have to perform without any marked diminution of strength, or without rigidity or rapidity of movements, and who died from an acute inflammation elsewhere than in the brain. In this case it was found that the little finger could not pass through the foramen magnum, so that the medulla oblongata must have been reduced to a small number of fibers, and the parts must have been in a very altered state. Still that man had shown no symptom of paralysis, no symptom of great disturbance of any kind. There are not many such cases ; but there are, at least, to my knowledge, three or four. There are a good many cases in which the odontoid process had been displaced, and pressing upon the medulla oblongata, had reduced a great part of its size. In these cases hardly any paralysis had been noticed. There are other cases, such as fracture of the occipital bone, displacing a part of that bone, and squeezing the medulla oblongata so as to reduce it to a small number of fibers. In other cases, as one reported by Dr. John W. Ogle, of London, two tumors, both attached to the foramen magnum, the one on one side and the other on the other, had squeezed the medulla oblongata, and reduced it to a small volume indeed, and without any considerable paralysis. In fact there are many cases, in which either by outside pressure or by disease in that organ, the medulla has been greatly reduced, and no marked paralysis has been observed. In one case, reported by Dr. Abercrombie, the paralysis observed was exceedingly slight, and still Dr. Abercrombie states that an abscess occupied the whole diameter of the medulla oblongata ; what the diameter was, unfortunately, he does not say. There was a considerable lesion, but very slight paralysis. It is extremely remarkable that the medulla oblongata, which, as you know, cannot be pricked without death being produced, can, however, in many cases, stand considerable alteration, considerable pressure, without any marked paralysis occurring.

I might say something as regards other parts of the base of the brain ; and the argument would certainly derive force from the details of these facts ; but unfortunately, for some reason, I have not been able to obtain the details. I hope it will be sufficient for me to state that there are a great many cases of considerable destruction of these various parts of the base of the brain, which extend from the lower part of the medulla oblongata up to a part of the crura cerebri in the two hemispheres, without any marked paralysis, or with paralysis quite inadequate to the lesion that exists. We must therefore draw the conclusion from these facts, that relatively a few fibers may be quite sufficient to establish communication between the brain and the muscles.

I pass now to consider a point relating to the motor centers of the brain. I said yesterday that I could not admit in the presence of the facts which are in my possession, that there are clusters or agglomerations of cells in certain parts of the brain which are employed in a peculiar function as regards a movement. You know it is now admitted that both in front and behind the fissure of Rolando, there are cells employed in moving the arm on the opposite side. I will not discuss the facts which bear directly against admitting such view. I have already cited a good many facts in opposition to such a view. What I have to do now is to show that view which I consider as correct—that which I proposed, in a lecture in Boston some few years ago. That view consists in admitting that there are cells employed in the same functions scattered all over the brain, so that any part of the brain can be destroyed without any loss of any of the functions of that organ. These cells certainly must be united one with another by fibers; and such union must take place also, even if you have all the cells agglomerated together. There must be communication between them for concert, for harmony in action; so that the same necessity exists in this view of my own as well as in that which I reject. It makes no difference whatever, whether the fibers that establish that communication are only one line or the hundredth part of a line, or two inches in extent, as of necessity any communication is independent of length of fiber for its effects, as Prof. Flourens, of Paris, long ago, established. These facts have been too much neglected lately by experimenters who try to establish the new views concerning the existence of clusters of cells—no doubt of certain limited functions. If you take away the brain by layers, beginning with the front part, you can go until you take away almost all of the two hemispheres without any marked effect upon movements; and still very suddenly you will find paralysis appearing. That paralysis may be complete as soon as it appears, although you may have taken away only a few cells more. If you repeat the experiment, by proceeding from the back part of the brain towards the front, you will have very much the same result. In fact, the question is a question of quantity of brain-matter, and not a question as to the function existing in one part. The demonstration given long ago by Prof. Flourens ought to have satisfied physiologists. I remember, in my younger days when I began to lecture, I repeated the experiment a great many times and found it correct. Since that time I have seen no reason to change my opinion. What, then, can we say in the presence of such facts? Certainly they demonstrate that the function does not belong to a cluster of cells anywhere, but belongs to cells scattered everywhere, so that any part of the brain can be taken away without loss of function. Pathological facts in man bear out the same thing. When we find the anterior lobes have been destroyed by fracture, taking away a large part of, or by any disease destroying, the tissue, all such cases very different from each other, but all having some vessels either completely destroyed, or taken away, or extremely altered, there was either no paralysis or paralysis inadequate to the lesion. I do not mean to say that there is never paralysis in these cases. On the contrary it exists sometimes; and indeed disease located in some part of the anterior lobes can produce paralysis. But there are a great many cases in which the destruction may involve the two anterior lobes without making paralysis. In the same way, although in a smaller number of cases, destruction of the two middle lobes has been observed without any marked paralysis.

I need not say that it has been observed that destruction of the two posterior lobes has also existed without paralysis. I need not say that no physiologist now feels inclined to admit that the posterior lobes of the brain are employed in any way whatever in movements of the body. But the very view that the posterior lobes are not employed, according to modern physiologists, for moving any part of the body, is in opposition to a number of facts quite different from those I have mentioned, in which a lesion of a small extent in the posterior lobes has produced paralysis; so that degeneration or destruction on the two sides of the brain, of considerable size, may not be followed by paralysis, while a lesion of a small extent in these two parts can produce paralysis. We are entitled, therefore, to look upon paralysis as something different from what it is admitted to be. Indeed, it is shown from the facts we have presented, that there is no part of the brain, which sometimes being destroyed, has not allowed the persistence of voluntary movement. In the presence of such facts, we must admit that cells employed to move any part of the body are scattered about the brain.

The next point that I wish to establish is with regard to what is called the *clavier theory.* This is the theory according to which communication in a direct way takes place between the brain and muscles. As regards this view, however, the number of facts against it is immense. The theory, at present, has been modified. Originally it was admitted that the fibers which move a given muscle must all be extended like wires to the muscle through the spinal cord and cerebral lobes. It is exactly, in part at least, the very same theory which is now admitted by Terris and other leading physiologists. This theory now is that the pretended centers which are placed in the middle lobe, and in a very limited part of the anterior lobe, are connected by fibers with the corpus striatum, and also by some fibers with the crura cerebri, chiefly in their anterior part, to continue downwards in the pons varolii or in the medulla oblongata, occupying, according to certain physiologists, the anterior pyramids, and according to others, the lateral columns of the medulla oblongata. So that the theory is pretty much the same as it was long ago, excepting that most of these fibers are now recognized to pass from cells, and not to go directly from the brain and muscles without any communication with the cells. It makes no difference whether there are cells or not. The same arguments, brought long ago against the *clavier* theory, are good against the new one. It is impossible to admit the view by reason of the facts already presented. I need not repeat them. We find the two crura cerebri destroyed in the anterior part without marked paralysis; we find the two corpora striata almost completely destroyed without marked paralysis. According to the *clavier* theory, when the will wishes to produce a certain movement, it has but to strike a key, which acts upon certain fibers, producing movement. No such thing certainly exists. When we see the medulla oblongata so reduced that only about one-fifth remains, such cases certainly leave no doubt that such transmission of nerve currents between the brain and the muscles does not exist. It is certain that we must admit that a few fibers are sufficient; and if so, it leads to the necessity of admitting something else than mere nerve current.

We find not only from these facts, but from disease of the spinal cord, that a something like a message must be sent through the few wires that remain in cases of considerable lesion in the brain and spinal cord in voluntary movements. Through these few fibers the will can transmit a message; but there is no possibility, that I can see, of understanding that these few fibers may be sufficient to transmit direct action to the muscles. You know our voluntary movements are not voluntary all the time—that if we wish to walk from here to a place ten feet in front of us, we give the order in a clear way, as otherwise our movements will not be executed just as we wish. We must unconsciously give the end to be reached. We do not give any other order but mention the end, and when the machinery has been set at work the movements will continue. The power of will in these cases is exceedingly limited. The power of the will consists only in giving the order to reach the end. The execution is performed by the parts located in the spinal cord, from the nerves which come from the base of the brain, and are located in the *medulla oblongata.*

If we understood that it is only an order which is given to reach an end, or to execute a certain thing, there is no need of a great number of fibers. The order is not given by a constant transmission of a current all the time. The order we give once for all, and if we try to interfere with the movements of our limbs which we are working, we do not execute the movement well. In every movement that we execute, either with the fingers, when we play the piano, or any movement of any kind executing anything that requires exactitude in movement and also in reaching an end—in all these cases if we interfere we become clumsy. If we look at our legs when we walk, if we try to ameliorate our steps, we become extremely clumsy and do not walk as well as when we do not pay attention at all to the order to move. So it is when we want to shoot with a gun. If we pay attention to the movements of the fingers, and if we do not pay attention only to the end, we are sure to miss the object. So that the great point is to understand the end clearly, and let the mechanism execute what is to be done to reach that end. If so, when we move, when we execute a series of movements, it is not a series of nerve currents coming from the brain to the muscles which takes place. An order given implies a message sent. But if communication can take place between cells; if cells are able to understand a message; if cells are endowed with power similar to the power in the brain in the act of thinking, it is quite easy to understand that a few fibers are perfectly sufficient. But if we do not admit that it is from a message that we have an order to the muscles, I am at a loss to understand how a few fibers will be sufficient for the transmission of the orders of the will to the muscles.

To-morrow I intend to examine at length the diagnosis of paralysis and convulsions in the various parts of the base of the brain, and come also to that most important part of this course of lectures, although it will be rather a short one—that is, the therapeutics of the nervous system, as we can ground it now on the new theories I have tried to bring forward.

---

Evidences of Medical Activity in the United States found in Foreign Journals.—Time was when medical authors and medical treatises of American origin were greeted on the other side of the water with contemptuous disdain. It is curious to note the constantly increasing change in this particular. For example, the latest issue of the venerable and conservative *Edinburgh Medical Journal* contains an original communication from an American author; five of its ten book reviews relate to American publications, all favorably criticized; and in the Periscope, which embraces 16 items there are nine credited to American Medical journals.

Claude Bernard the great physiologist, died at Paris, on February 11th.

